# Phosphoproteome Study of *Escherichia coli* Devoid of Ser/Thr Kinase YeaG During the Metabolic Shift From Glucose to Malate

**DOI:** 10.3389/fmicb.2021.657562

**Published:** 2021-04-06

**Authors:** Abida Sultan, Carsten Jers, Tariq A. Ganief, Lei Shi, Meriem Senissar, Julie Bonne Køhler, Boris Macek, Ivan Mijakovic

**Affiliations:** ^1^Novo Nordisk Foundation Center for Biosustainability, Technical University of Denmark, Kongens Lyngby, Denmark; ^2^Quantitative Proteomics and Proteome Center Tübingen, Interfaculty Institute for Cell Biology, University of Tübingen, Tübingen, Germany; ^3^Systems and Synthetic Biology Division, Department of Biology and Biological Engineering, Chalmers University of Technology, Gothenburg, Sweden

**Keywords:** phosphoproteome, protein kinase, SILAC, metabolic adaptation, kinase-substrate relationship

## Abstract

Understanding phosphorylation-mediated regulation of metabolic enzymes, pathways, and cell phenotypes under metabolic shifts represents a major challenge. The kinases associated with most phosphorylation sites and the link between phosphorylation and enzyme activity remain unknown. In this study, we performed stable isotope labeling by amino acids in cell culture (SILAC)-based proteome and phosphoproteome analysis of *Escherichia coli* Δ*yeaG*, a strain lacking a poorly characterized serine/threonine kinase YeaG, to decipher kinase-substrate interactions and the effects on metabolic phenotype during shifts from glucose to malate. The starting point of our analysis was the identification of physiological conditions under which Δ*yeaG* exhibits a clear phenotype. By metabolic profiling, we discovered that Δ*yeaG* strain has a significantly shorter lag phase than the wild type during metabolic shift from glucose to malate. Under those conditions, our SILAC analysis revealed several proteins that were differentially phosphorylated in the Δ*yeaG* strain. By focusing on metabolic enzymes potentially involved in central carbon metabolism, we narrowed down our search for putative YeaG substrates and identified isocitrate lyase AceA as the direct substrate of YeaG. YeaG was capable of phosphorylating AceA *in vitro* only in the presence of malate, suggesting that this phosphorylation event is indeed relevant for glucose to malate shift. There is currently not enough evidence to firmly establish the exact mechanism of this newly observed regulatory phenomenon. However, our study clearly exemplifies the usefulness of SILAC-based approaches in identifying proteins kinase substrates, when applied in physiological conditions relevant for the activity of the protein kinase in question.

## Introduction

Metabolic adaptation is one of the major bacterial responses for coping with the changing environment (Liebeke and Lalk, 2014; Goo et al., 2015). By rearranging their metabolic functions, bacteria can resist antibiotic treatments and evade the host immune response during infection (Sprenger et al., 2018; Jiang et al., 2019), they can adapt to severe hypoxia or starvation (Rittershaus et al., 2018; Gray et al., 2019), or they can effectively switch between alternative carbon or nitrogen sources as they become available (Deutscher, 2008; Esteves-Ferreira et al., 2018). Metabolic adaptation commonly involves changes in transcription of genes encoding metabolic enzymes, an approach that is costly both in terms of time and resources (requirement of building blocks, ATP, etc.; Chubukov et al., 2013). Another, faster mechanism of response is the modification of activity of enzymes already present in the bacterial cell. This is typically achieved by allosteric regulation (Xu et al., 2012; Link et al., 2013) or reversible post-translational modifications (PTMs; Nussinov et al., 2012; Macek et al., 2019) of bacterial proteins.

Among various PTMs known to regulate bacterial metabolism, protein phosphorylation is arguably the most extensively characterized. Phosphorylation and dephosphorylation are a metabolic switch mediated by kinases and phosphatases. Many examples of protein phosphorylation affecting the expression of metabolic genes are known. Notably, bacterial metabolic adaptation processes are known to depend on histidine-kinases and response regulators of the so-called two component systems (Charbonnier et al., 2017; Quiroz-Rocha et al., 2017; Gómez-Mejia et al., 2018). Herein, transient autophosphorylation of bacterial histidine kinases on histidine residues and response regulators on aspartate residues leads to altered expression of metabolic operons. Carbon catabolite repression, a major metabolic switch in bacteria, is known to critically depend on histidine and cysteine phosphorylation of components of the phosphoenolpyruvate:carbohydrate phosphotransferase system (Deutscher et al., 2014). In Firmicutes, carbon catabolite repression is also regulated by serine-phosphorylation of the major transcriptional regulator CcpA (Schumacher et al., 2004). Bacterial protein-tyrosine and -serine/threonine kinases are known to regulate the metabolism by directly phosphorylating transcription regulators and modifying their affinity for binding of target DNA sequences (Derouiche et al., 2013, 2015; Kobir et al., 2014).

While protein phosphorylation is known to play a major role in transcriptional regulation, hitherto, direct regulation of enzyme activity by serine/threonine phosphorylation has been reported for only a handful of bacterial metabolic enzymes. One well-studied example is phosphorylation of isocitrate dehydrogenase (Icd), which controls the branching point between the glyoxylate shunt and the TCA cycle. In *Escherichia coli*, isocitrate dehydrogenase kinase/phosphatase AceK phosphorylates Icd on a serine residue, thereby inactivating the enzyme and directing the carbon flux through the glyoxylate shunt (LaPorte and Chung, 1985; Thorsness and Koshland, 1987). In the pathogen *Mycobacterium tuberculosis*, the S-adenosyl-L-homo-cysteine hydrolase (SahH), which plays an important role in regulating cellular methylation processes, gets inactivated by serine/threonine phosphorylation (Corrales et al., 2013). Inhibition of SahH activity by phosphorylation is thought to act by decreasing affinity toward the essential cofactor NAD^+^ (Singhal et al., 2013). In the same bacterium, phosphorylation of protein GarA abolishes its direct inhibition of metabolic enzymes: α-ketoglutarate dehydrogenase, glutamate dehydrogenase, and glutamate synthase (Ventura et al., 2013). A study by Brunk et al. (2018) demonstrated how phosphorylation-mediated regulation of *E. coli* enzymes, such as enolase, transaldolase, and serine hydroxymethyltranferase, impacts metabolic pathways and cellular fitness in a changing environment. Virmani et al. (2019) also reported regulation of enolase by Ser/Thr kinase PrkC in sporulating *Bacillus anthracis*. PrkC was found to augment the germination process by maintaining enolase quantity/expression and activity. In *E. coli* and *Bacillus subtilis*, protein-tyrosine kinase-mediated phosphorylation of UDP-glucose dehydrogenases is known to increase their metabolic activity by increasing their affinity for NAD^+^ (Mijakovic et al., 2003; Lacour et al., 2008; Petranovic et al., 2009). The *M. tuberculosis* Ser/Thr protein kinase PknJ was shown to phosphorylate the rate-limiting enzyme of glycolysis pyruvate kinase A (Arora et al., 2010). Interestingly, bacterial DNA metabolism is also regulated by direct phosphorylation of the recombinase RecA, demonstrated in *B. subtilis* (Bidnenko et al., 2013) and *Deinococcus radiodurans* (Rajpurohit et al., 2016).

Despite there being relatively few cases of well-characterized regulation of bacterial metabolic enzymes *via* phosphorylation, there is plenty of evidence that metabolic enzymes are extensively phosphorylated in bacterial cells. From the publications of the very first site-specific bacterial phosphoproteomes (Macek et al., 2007, 2008), it became apparent that protein phosphorylation is specifically enriched in metabolic enzymes (Soufi et al., 2008). To the point, a study by Hansen et al. (2013) identified 512 tyrosine-phosphorylation sites on 342 proteins in *E. coli* K12 MG1655 with roles in glycolysis and the TCA cycle, including fructose-1,6-biphosphate aldolase A and B, glyceraldehyde-3-phosphate dehydrogenase, phosphoglycerate kinase, enolase, isocitrate lyase, and fumarase. Similarly, phosphoproteome analysis of *Streptococcus suis* Δ*stk* (Ser/Thr kinase deletion) identified 12 phosphoproteins with differential phosphorylation levels, where glycolytic enzymes FBA, GADPH, and translation associated EF-Tu were found upregulated, and the remaining proteins involved in cell division and purine metabolism were downregulated in phosphorylation levels (Zhang et al., 2017). However, putative phosphorylation-mediated regulation of these metabolic enzymes has not yet been addressed. In that sense, specific follow-up studies are required to establish any links that may exist between enzyme phosphorylation and activity.

One major challenge in establishing a link between enzyme phosphorylation and enzyme activity, or enzyme phosphorylation and the protein kinase that phosphorylates it, is the transient nature of protein phosphorylation. Therefore, as it has been argued before (Macek et al., 2019), it is essential to pick the correct physiological conditions when seeking to establish such links. In this study, we focused on an *E. coli* serine/threonine protein kinase YeaG (Figueira et al., 2015) that has not been extensively characterized and whose physiological substrates are unknown. As reported by Figueira et al. (2015), following sustained periods of nitrogen starvation, the wild type *E. coli* displayed metabolic heterogeneity with the metabolically active population displaying impaired viability. By contrast, the ∆*yeaG* mutant existed as a single metabolically active population. It is well established that bacteria promote survival or adaptation to sustained environmental adversity by generating phenotypically diverse subpopulations. The non-growing phenotype (persisters) typically neglects the available carbon source and enters a dormant state, causing a lag phase (van Heerden et al., 2014). It is recognized that under various stress conditions, *E. coli* and related bacteria typically increase the accumulation of RpoS sigma factor, which controls the general stress response and increases survival. The reduced lag phase observed for the ∆*yeaG* mutant in the study by Figueira et al. (2015), was ascribed to indirect transcriptional repression of the toxin-antitoxin modules *mqsR*/*mqsA* and *dinJ*/*yafQ*, which in turn positively impacts RpoS transcription and translation.

In order to find physiological substrates of YeaG and hopefully link them to cellular regulation, we focused on growth conditions where ∆*yeaG* strain has a significant growth phenotype. Given the known involvement of YeaG in metabolic adaptation, we expanded the phenotypic profiling of the ∆*yeaG* strain focusing on various carbon source shifts. The most striking phenotype was shortening the lag phase in transition from growth on glucose to growth on malate. Focusing on this phenotype, we compared quantitative proteome and phosphoproteome data of ∆*yeaG* and wild type strains after the shift from glucose to malate, to identify differentially expressed and phosphorylated proteins. By using *in vitro* phosphorylation assays, we identified isocitrate lyase AceA as the substrate of YeaG. Interestingly, YeaG was capable of phosphorylating AceA only in the presence of malate. Given the known negative impact of AceA on short-term adaptation to glucose starvation (Maharjan et al., 2005), it is probable that YeaG-dependent phosphorylation contributes to this negative effect and is at least in part responsible for the lag phase phenotype of ∆*yeaG*.

## Materials and Methods

### Reagents and Oligonucleotides

Plasmid and PCR purification kits were purchased from Macherey-Nagel (AH Diagnostics, Aarhus, Denmark). Oligonucleotide primers (Table 1) were purchased from Integrated DNA Technologies (IDT, Leuven, Belgium) and Macrogen (Amsterdam, Holland). Unless specified all the chemicals were purchased from Sigma-Aldrich (Copenhagen, Denmark).

**Table 1:** List of oligonucleotides used in this study.

Name	Sequence	Description
yeaG colPCR fwd	CAGTTTACCTCTTCCGGGAG	KO fragment
yeaG colPCR rev	CAATAAACCAGGTCATATGCCCC	KO fragment
His-yeaG_USER_fwd	ATATACCAUATGCATCATCATCATCATCACAATATATTCGATCACTATCGCCAG	USER cloning, yeaG 5'
His-yeaG_USER_rev	ATGCTAGTUAAGACGATTTACGTACGCGC	USER cloning, yeaG 3'
pET28a_USER_fwd	AACTAGCAUAACCCCTTGGG	USER cloning, pET28a
pET28a_USER_rev	ATGGTATAUCTCCTTCTTAAAGTTAAAC	USER cloning, pET28a
yeaG_EcoRI_F	TAGGTCTCGAATTCATTAAAGAGGAGAAATTAACTATGAATATATTCGATCACTATCGCCAG	EcoRI, yeaG 5'
yeaG-His_SacI_R	ATATGAGCTCTTAGTGATGGTGATGGTGATGAGACGATTTACGTACGCGC	SacI, 6xHis, and yeaG 3'
aceA_BamHI_F	ATATGGATCCATGAAAACCCGTACACAACAAATTG	BamHI, AceA 5'
aceA_SacI_R	ATATGAGCTCTTAGAACTGCGATTCTTCAGTGG	SacI, AceA 3'
acnB BamHI fwd	ATATGGATCCATGCTAGAAGAATACCGTAAGCAC	BamHI, AcnB 5'
acnB SacI rev	ATATGAGCTCTTAAACCGCAGTCTGGAAAATC	SacI, AcnB 3'
ppsA BamHI fwd	ATATGGATCCATGTCCAACAATGGCTCG	BamHI, PpsA 5'
ppsA SacI rev	ATATGAGCTCTTATTTCTTCAGTTCAGCCAGG	SacI, PpsA 3'
sodB BamHI fwd	ATATGGATCCATGTCATTCGAATTACCTGCAC	BamHI, SodB 5'
sodB SacI rev	ATATGAGCTCTTATGCAGCGAGATTTTTCG	SacI, SodB 3'

Restriction enzyme sites are underlined.

### Construction of Gene Deletion Strains

Deletions of genes encoding protein kinases were constructed in *E. coli* K12 MG1655 (genotype: F ¯ λ̄ *ilvG rfb*-50 *rph*-1) using *λ* Red-mediated recombination with temperature sensitive plasmid pSIJ8 (arabinose inducible *λ* Red recombineering genes and rhamnose inducible flippase recombinase; Datsenko and Wanner, 2000; Thomason et al., 2014). Linear DNA fragments containing a kanamycin resistance gene flanked by FLP recognition target (FRT) sites were amplified from strains in the Keio collection (Baba et al., 2006) by PCR (oligonucleotides are listed in Table 1). The PCR products were introduced by electroporation into cells expressing *λ* Red by either elevated temperature induction at 42°C or addition of 0.2% L-arabinose, at OD_600_ of 0.2 at 30°C. Recombinants with insertion in the correct location were confirmed by colony PCR. The plasmid pSIJ8 was cured from cells by incubation at 37°C on plates.

### Screening of Growth During Metabolic Adaptation

For growth screening experiment, *E. coli* cells were cultured in complete (LB and Neidhardt EZ) and minimal M9 media [12.8 g/L Na_2_HPO_4_·7H_2_O, 3.0 g/L KH_2_PO_4_, 0.5 g/L NaCl, 1.0 g/L NH_4_Cl, 2 mM MgSO_4_, 0.1 mM CaCl_2_, and 2000X dilution of trace elements solution (3 g/L FeSO_4_·7H_2_O, 4.5 g/L ZnSO_4_·7H_2_O, 0.2 g/L CuSO_4_·2H_2_O, 0.7 g/L MnCl_2_·4H_2_O, 0.3 g/L CoCl_2_·6H_2_O, 0.4 g/L Na_2_MoO_4_·2H_2_O, 4.5 g/L CaCl_2_·2H_2_O, 15 g/L EDTA, 1 g/L H_3_BO_3_, and 0.1 g/L KI)] supplemented with various carbon sources and stressors (i.e., 0.4% glucose, 0.3% malate, 0.4% fructose, 0.4% sorbitol, 0.4% pyruvate, 0.4% succinate, 5 mM EDTA, and 0.5 M NaCl). Briefly, starter cultures of each strain were inoculated from single colonies from agar plates into 300 μl of M9 medium with 0.4% glucose in 96 deepwell plates. Biological triplicates derived from different colonies were cultivated for each strain at 37°C with 300 rpm shaking. After 14–16 h, wells of a 96-well microtiter plates (Enzyscreen) containing M9 medium with 0.4% glucose (total volume 300 μl) were inoculated to an initial OD_600_ of 0.03 and incubated in a Growth Profiler 960 micro-bioreactor system (System Duetz, EnzyScreen, Heemstede, Netherlands) at 37°C with 250 rpm shaking. When the cultures reached an OD_600_ of 1, aliquots of cell culture were transferred to fresh M9 medium supplemented with different carbon sources and incubated at 37°C with 250 rpm shaking. Growth was monitored every 20 min by optimal image scanning, and integrated G-values were converted into equivalent OD_600_ values using the image analysis software (Enzyscreen).

### Cell Cultivation and Protein Extraction for Mass Spectrometry Proteomics Analysis

For phosphoproteomics experiments, M9 medium was supplemented with either unlabeled 0.025% “light” L-lysine (^12^C_6_^14^N_2_-lysine, Lys0), or labeled “medium” L-lysine (^2^H_4_-lysine, Lys4) and “heavy” L-lysine (^13^C_6_^15^N_2_-lysine, Lys8; Cambridge Isotope Laboratories, Andover, MA). Wild type *E. coli* K12 and Δ*yeaG* were cultured in stable isotope labeling by amino acids in cell culture (SILAC) medium supplemented with 0.4% glucose at 37°C to an OD_600_ of 1.0 and shifted to fresh SILAC medium with either 0.4% glucose or 0.3% malate. After reaching an OD_600_ of 0.6, the cells were harvested by centrifugation at 7,500 g for 10 min at 4°C and snap-frozen in liquid nitrogen. Cells were lysed in sodium dodecyl sulphate (SDS)-buffer (4% SDS, 100 mM Tris-HCl pH 8.0, and 1 mM EDTA) supplemented with protease inhibitors (Complete protease inhibitor cocktail tablets, Roche Diagnostics) and phosphatase inhibitors (5 mM β-glycerophosphate, 5 mM sodium fluoride, and 1 mM sodium vanadate) for 10 min at room temperature, followed by sonication for 1 min at an amplitude of 40% (pulses of 2 s on and 2 s off). Cell debris was removed by centrifugation at 16,000 *g* for 30 min. Lysates were precipitated in chloroform/methanol and subsequently re-dissolved in denaturation buffer (6 M urea, 2 M thiourea in 10 mM Tris-HCl, pH 8.0). Protein concentration was estimated by Bradford assay (Bio-Rad). Equal amounts (1:1:1) of protein from the differentially SILAC-encoded cells were pooled for subsequent mass spectrometry (MS) sample preparation. Triple SILAC experiments were performed with wild type grown in M9 medium with glucose labeled with “light” lysine as the common point for integration of all experiments. Biological duplicates derived from different colonies were used for each strain.

### MS Sample Preparation

To determine complete incorporation of labeled amino acids, an aliquot of 10 μg of medium and heavy labeled protein extracts were digested (as described below) and analyzed on the mass spectrometer. Incorporation of 94% and above was accepted and used for further analysis (Supplementary Figure 1). Four milligrams of protein from each SILAC condition was mixed in a 1:1:1 ratio to a total of 12 mg. A mixing check was performed to check the mixing ratio. Samples were then subjected to in-solution digestion as described previously, with few modifications (Macek et al., 2007). Briefly, protein disulfides were reduced with 1 mM dithiothreitol (DTT) for 1 h at room temperature, and subsequently alkylated with 5.5 mM iodoacetamide (IAA) for 1 h at room temperature in the dark. Proteins were digested with endoproteinase Lys-C (Wako) in an enzyme:protein ratio of 1:100 (w/w) for 3 h, followed by a 4-fold dilution with water, and further digestion overnight with Lys-C 1:100 (w/w). Protease activity was quenched by acidification with trifluoroacetic acid (TFA) to a final concentration of 1–2%, and the resulting peptide mixture was desalted and concentrated on reverse-phase Sep-Pak C18 Cartridge (Waters). Peptides were eluted with 80% acetonitrile (ACN) and 6% TFA. The ACN was removed by vacuum centrifugation for 30 min, and peptide concentration was estimated by measuring the absorbance at 280 nm (Nanodrop 2000C, Thermo Scientific).

### Phosphopeptide Enrichment Using TiO_2_ Beads

An aliquot of the peptide mixture was used for proteome analysis, while the remaining sample was enriched for phosphopeptides using titanium dioxide beads (TiO_2,_ 5 μm Titansphere, GL Sciences, Japan). The TiO_2_ beads were washed with 80% ACN and 6% TFA prior to incubation with the peptide mixture to a 10:1 peptide-to-beads ratio, with gentle rotation for 10 min. After incubation, the beads were spun down, and the supernatants were incubated with fresh TiO_2_ beads for a second enrichment round. The procedure was repeated for five consecutive rounds. Beads were loaded onto in-house packed C_8_ StageTips and washed with 80% ACN and 6% TFA. The phosphopeptides were eluted with 2 × 50 μl 20% NH_4_OH, pH 10.5, in 60% ACN, and acidified with 20 μl of 20% TFA. Eluted peptides were concentrated and ACN evaporated and loaded onto C_18_ StageTips (Rappsilber et al., 2007).

### Analysis Using Nano-Flow LC-MS/MS

Samples were analyzed on an EASy-nanoLC 1,000 (Proxeon, Thermo Scientific) coupled to a Q-Exactive orbitrap HF instrument (Thermo Scientific). Peptides were separated on a 15 cm column (75 μm inner diameter) in-house packed with 1.9 μm reverse-phase C_18_ beads (Reprosil-Pur, Dr. Maisch, Germany) with a maintained column temperature of 40°C. Phosphopeptides were separated over a 49 min gradient ranging from 10 to 33% of the separation buffer (80% ACN and 0.1% formic acid) in 43 min, ramped to 50% B in 3 min, and additionally to 90% B for 3 min at a flow rate of 200 nl/min, followed by a washout at 90% B for 8 min. Protein samples spiked with peptide mixtures (UPS2, Sigma-Aldrich) were separated using a 120 min gradient of 10–30% B. The Q Exactive HF was operated using Xcalibur v. 2.2 in the data dependent mode to automatically switch between full scan MS and MS/MS acquisition as described previously (Michalski et al., 2011). Spray voltage was set to 2 kV, s-lens RF level at 50, and heated capillary temperature at 275°C. Survey full-scans for the MS spectra (300–1,650 m/z) were acquired at a resolution of 120,000 with an ion target value of 3E6 charges with a maximum fill time of 25 ms. Higher-energy collisional dissociation (HCD) fragment scans were acquired with optimal setting for parallel acquisition, using 1.4 m/z isolation width and normalized collision energy of 27. For proteome, a top12 method was employed with fragment scan resolution of 30,000 at 200 m/z and target value of 1E5 with maximum fill time of 45 ms. For phosphoproteomes, a top7 method was employed with fragment scan resolution of 60,000 at 200 m/z and target value of 1E5 with maximum fill time of 120 ms.

### MS Data Processing and Analysis

The acquired raw data files were processed by MaxQuant v1.6.0.0 and searched against a target-decoy database of *E. coli* K12 MG1655 (taxonomy ID 83333, last modified November 6, 2016) containing 4,306 protein entries using the integrated Andromeda search engine (Elias and Gygi, 2007; Cox et al., 2011). Carbamidomethylation of cysteines was specified as fixed modification for both protein and phospho-enriched samples. Variable modifications considered were oxidation of methionine and protein N-terminal acetylation. The phospho-enriched dataset was searched using phosphorylation of Ser, Thr, and Tyr residues as variable modification. A false discovery rate (FDR) was set to 1% for both protein and phospho-site, and a minimum peptide length of six amino acids was required. Spectra were searched with a mass tolerance of 6 ppm for precursor ions and 20 ppm for fragment ions. Endoproteinase Lys-C was selected as protease and a maximum of two missed cleavage sites as allowed.

### Functional Enrichment Analysis

Gene Ontology (GO) annotation of *E. coli* K12 MG1655 was retrieved from Uniprot-GOA (downloaded 02.06.2018). PANTHER Classification system was used to perform GO term characterization and enrichment analysis according to GO categories for cellular compartment, biological process, and molecular function. Enrichment/fold change analysis of GO categories was done using the Fisher’s exact test. All the GO terms, which were significant with *p* < 0.05 after correcting for multiple testing by Benferroni FDR, were selected as over- or under-represented.

### Expression and Purification of YeaG and Its Putative Substrates

The genes encoding potential YeaG substrates were amplified from genomic DNA of *E. coli* K12 MG1655 using specific primers with appropriate restriction sites listed in Table 1. All PCR fragments were ligated in the appropriate restriction sites of pQE30 vector (Qiagen) and propagated in *E. coli* NM522. After verification of the inserts, the resulting plasmid DNA was used to transform *E. coli* M15. Cultures were grown in LB medium supplemented with 100 μg/ml ampicillin and 25 μg/ml kanamycin at 37°C with vigorous shaking. Protein expression was induced with addition of 1 mM isopropyl β–D-thiogalactopyranoside (IPTG) at an OD_600_ of 0.6 and cells were harvested 3 h after induction. Cells were lysed in lysis buffer (50 mM Tris-HCl pH 7.5, 100 mM NaCl, and 10% glycerol) supplemented with 1 mg/ml lysozyme and 5 μg/ml DNase, followed by sonication (2 min with 2 s on and 2 s off at 30% amplitude) and centrifugation (20,000 *g* for 20 min). Proteins were purified using nickel-nitrilotriacetic acid (Ni-NTA) beads (Ni-NTA superflow, Qiagen or His HF nickel affinity gel, Sigma-Aldrich) according to the manufacturer’s instructions and desalted on PD-10 columns (GE Healthcare). Proteins were separated by electrophoresis on 12% SDS-polyacrylamide gels and their concentration was estimated by Bradford assay (Bio-Rad).

### Western Blotting

For each sample, 5 μg of protein was separated on a 4–20% Tris-glycine SDS polyacrylamide gel and transferred onto polyvinylidene difluoride membrane using the iBlot dry blotting system (Invitrogen). The membrane was blocked for 2 h with TBST (10 mM Tris, 150 mM NaCl, 0.1% Tween20, and pH 7.6) containing 5% BSA, washed five times with TBST for 5 min, and probed with appropriate antibody from rabbit/mouse. Anti-phospho-serine antibodies were from Cell Signaling Technology and anti-phospho-threonine antibodies were from Abcam. Both antibodies were used according to the manufacturer’s recommendations. Subsequently, goat anti-rabbit/mouse coupled to horseradish peroxidase antibody (5000x dilution, Bio-Rad) was used and detected using the ECL prime Western blotting detection reagent (GE Healthcare). Exposure time for the film varied between 1 and 5 min.

### *In vitro* Protein Phosphorylation Assay

For *in vitro* phosphorylation assays, 6 μg YeaG and 5 μg AceA were incubated at 37°C for 2 h in a reaction containing 50 mM HEPES pH 7.4, 10 mM MgCl_2_, 10 mM MnCl_2_, 100 mM KCl, 10 mM ATP, and 0.1% triton X-100, with or without 10 mM malate. Reactions were started by adding ATP and stopped by adding sample buffer for sodium dodecyl sulphate–polyacrylamide gel electrophoresis (SDS-PAGE; Bio-Rad) and boiling at 100°C. Samples were separated on 10% SDS-PAGE copolymerized with and without Phos-tag acrylamide (Fujifilm). To prepare SDS-PAGE copolymerized with Phos-tag acrylamide, 50 μM Phos-tag acrylamide and 100 μM MnCl_2_ in final concentration were added into resolving gel. Protein bands were revealed by Coomassie brilliant blue R-250 staining.

## Results and Discussion

### Inactivation of YeaG Reduces Lag Phase During Metabolic Shift From Glucose to Malate

**Figure 1:**
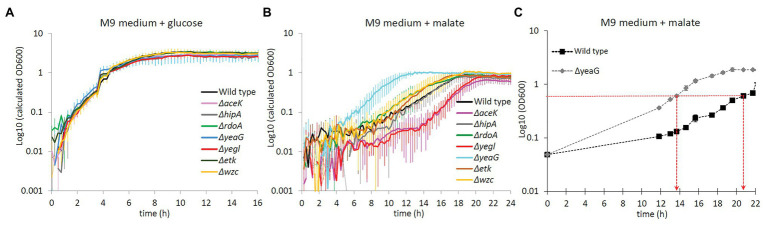
Growth phenotype of Δ*yeaG* during glucose to malate shift. **(A)** Growth profiler experiment for different *Escherichia coli* strains (names indicated in the figure inset) shifted from M9 medium supplemented with glucose to fresh M9 medium supplemented with glucose: control with no metabolic shift (average of biological triplicates). **(B)** Growth profiler experiment for different *E. coli* strains (names indicated in the figure inset) shifted from M9 medium supplemented with glucose to fresh M9 medium supplemented with malate: metabolic shift from glucose to malate (average of biological triplicates). **(C)** Growth profile of Δ*yeaG* and wild type strains in batch cultures scaled up for the stable isotope labeling by amino acids in cell culture (SILAC) proteomics experiment (average of biological duplicates). Time points when samples were collected for the MS proteomics analysis (at OD_600_ = 0.6) are indicated with red arrows.

YeaG has previously been reported to be involved in metabolic adaptation (Figueira et al., 2015). Hence, we compared the growth of the ∆*yeaG* strain to that of the wild type *E. coli* during metabolic shifts from glucose to various other carbon sources. *Escherichia coli* strains with individual inactivation of all other known serine/threonine/tyrosine kinases (∆*aceK*, ∆*hipA*, ∆*rdoA*, ∆*yegI*, ∆*etk*, and ∆*wzc*) were also used as controls. All the strains were initially grown in M9 medium with glucose as the sole carbon source and were then shifted to fresh medium with different carbon sources (fructose, sorbitol, malate, succinate, and pyruvate). Growth of all strains was monitored in an automated growth profiler. In the control experiment where cells were shifted from glucose to glucose, none of the kinase knockout strains exhibited a growth phenotype (Figure 1A). Among the conditions tested, the most striking phenotype of ∆*yeaG* was a significant reduction of the duration of lag phase upon transition from growth on glucose to growth on malate (Figure 1B). In order to investigate the mechanism by which YeaG controls the duration of metabolic adaptation to growth on malate, we decided to perform a quantitative MS (phospho)proteomics analysis with SILAC, comparing ∆*yeaG* to the wild type strain. To do this, we had to scale up the experiment to batch growth and supplement the growth media with different forms of lysine (Lys0, Lys4, and Lys8). In the scaled-up SILAC experiment growth set up, ∆*yeaG* strain retained its growth phenotype (Figure 1C).

### General Characteristics of the Detected ∆*yeaG* (Phospho)Proteome Upon Shift to Malate

Cell cultures (∆*yeaG* and wild type) labeled with the different forms of lysine (Lys0, Lys4, and Lys8) for SILAC labeling were initially grown on glucose. At an OD_600_ of 1, cells were shifted to malate as the sole carbon source, or back to glucose as a control experiment. After the shift, cell cultures were harvested when they reached an OD_600_ of 0.6 (late exponential phase). Proteins extracted from the corresponding SILAC cultures were combined in equal amounts and digested to peptides using endoprotease Lys-C. The wild type grown on glucose labeled with Lys0 was used as the common point in the two SILAC experiments, thus enabling integration of all datasets. In the first experiment, wild type shifted to glucose (Lys0) was combined with wild type shifted to malate (Lys4). In the second experiment, wild type shifted to glucose (Lys0) was combined with ∆*yeaG* shifted to glucose (Lys4) and ∆*yeaG* shifted to malate (Lys8). Incorporation level of the SILAC amino acids (Lys4 and Lys8) in all the samples was found to be |>94% (Supplementary Figure 1). Analysis of all the fractions (proteomes and phosphoproteomes) resulted in identification and quantification of 1,772 proteins (Supplementary Table 1). The estimated FDR was 0.29% at peptide level and 1.45% at protein level, respectively. Phosphoproteome analysis resulted in identification of 127 phosphorylation events (Supplementary Table 1). A majority of the localized phosphorylation events were observed on serine (67.7%), followed by threonine (28.31%) and tyrosine (3.94%). This agrees with a previous study in *E. coli* (Macek et al., 2008), which reported serine, threonine, and tyrosine phosphoproteome fractions of 67.9, 23.5, and 8.6%, respectively. Pearson correlation coefficients between the two biological replicates for differentially regulated proteome and phosphoproteome are provided in Supplementary material (Supplementary Figures 2, 3).

### Differences Between the Wild Type and ∆*yeaG* Proteome Upon Shift to Malate

**Figure 2:**
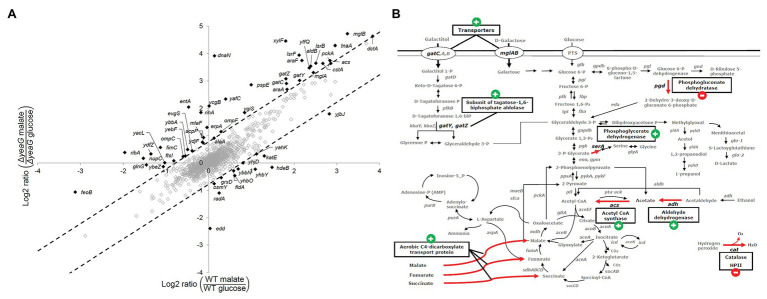
Differential expression of proteins during glucose to malate shift. **(A)** Plot of quantified protein ratios (in response to shift in carbon source from glucose to malate) for the Δ*yeaG* vs. the wild type strain. As a cutoff for assigning proteins differentially regulated in the two strains, we chose to use two SDs from the mean of the dataset (indicated by dotted lines). Proteins that were differentially upregulated or downregulated are marked in black and gene names are shown. **(B)** Proteins that are differently regulated during malate shift in the wild type and Δ*yeaG* strains mapped on the central carbon metabolism of *E. coli* K-12 MG1655. Red arrows are used to highlight metabolic and transport reactions catalyzed by the affected enzymes. The expression change during glucose to malate shift in Δ*yeaG* compared to the wild type strain is indicated next to the enzyme name by a plus/minus sign.

The SILAC approach enabled us to perform a quantitative comparison of the differentially expressed proteome between cells shifted from growth on glucose to a fresh glucose-containing medium and cells shifted from glucose to malate. A GO terms analysis of the differentially expressed proteome indicated that no specific category of biological processes, molecular functions, or cellular components was particularly affected in the ∆*yeaG* strain (Supplementary Figure 4). In Figure 2A, we attempted to visualize the main differences between ∆*yeaG* and the wild type strain proteomes during glucose to malate shift. On the “*y*” axis are expression ratios for individual proteins in ∆*yeaG* grown on malate over ∆*yeaG* grown on glucose – thus representing the expression change during glucose-to-malate shift. On the “*x*” axis, the same glucose-to-malate shift ratios are plotted for individual proteins in the wild type strain (a cutoff of two SDs from the dataset mean was applied). A large majority of proteins that are differentially expressed during the glucose-to-malate shift were not significantly affected by inactivation of YeaG. Among the proteins more strongly expressed in Δ*yeaG* upon shift to malate, there were proteins related to outer membrane-bound periplasmic space and monosaccharide transmembrane transport. In the wild type strain, cytosolic proteins related to stress response were more strongly expressed during shift to malate. In order to examine the possible impact of these proteome changes on the key phenotype of Δ*yeaG*, the reduced lag phase during glucose to malate shift, in Figure 2B, we mapped the changes in differential expression onto the map of *E. coli* central carbon metabolism. One category of proteins that was largely affected was the transporters. In particular, the kinase mutant overexpressed the aerobic C4 transporter DctA, capable of importing L-malate, alongside fumarate and succinate (Lukas et al., 2010). Stronger expression of DctA in Δ*yeaG* is consistent with the shorter lag phase observed for this strain during the shift to malate. Metabolic enzymes that were overexpressed only in the wild type during the shift included the 6-phospho-gluconate dehydratase, alcohol dehydrogenase, and catalase. Δ*yeaG* differentially overexpressed phosphoenolpyruvate carboxykinase, acetyl-CoA synthase, and aldehyde dehydrogenase. Based on the presented differences in the expressed proteome during glucose to malate shift, it seems plausible to presume that the YeaG protein kinase does not exert its metabolic control only by direct phosphorylation of target proteins/enzymes. It seems to also affect the expression of several genes, by an unknown mechanism that is not fully accounted for by the available literature. Bacterial serine/threonine kinases are known to phosphorylate transcription regulators and affect gene expression (Kalantari et al., 2015), so it is plausible to presume that YeaG might act by a similar mechanism.

### Inactivation of yeaG Affects Phosphorylation of Several Proteins During Glucose to Malate Shift

Since YeaG is a poorly characterized protein kinase (Figueira et al., 2015), we next asked what are the proteins that are differentially phosphorylated in the Δ*yeaG*. The SILAC approach has been previously used to quantitatively compare the phosphoproteome of a kinase knockout and wild type strain, in order to identify substrates of a protein kinase; proteins whose phosphorylation is attenuated in the kinase knockout (Shi et al., 2016). In this study, our approach was a bit more complex, since we compared the ratios of occupancy of individual phosphorylation sites during the glucose to malate shift, first individually for the wild type and Δ*yeaG* strains, and then made a strain-to-strain comparison. The results of differentially phosphorylated phosphorylation sites are shown in Table 2. Here, one can distinguish three scenarios. The first can be exemplified by the protein Hfq, phosphorylated at threonine 49. The occupancy of this phosphorylation site increases by almost the same factor during glucose to malate shift in the wild type and Δ*yeaG* strain. This means that YeaG exerts no influence, direct (phosphorylation), or indirect (e.g., affecting another kinase, which is directly responsible for phosphorylation) on the occupancy of this phosphorylation site. Another case, exemplified by phosphorylation of YjhQ at threonine 11, phosphorylation during glucose to malate shift is significantly reduced in the Δ*yeaG* strain compared to the wild type. Here, the most logical explanation is that YeaG is the kinase directly responsible for phosphorylating this amino acid. Having said that, other explanations are possible, e.g., an indirect effect, where YeaG activates another kinase, which is directly responsible for this phosphorylation. Therefore, further evidence would be required before YjhQ could be declared to be a direct substrate of YeaG. Finally, a third scenario can be observed, for example with phosphorylation of GlyA on tyrosine 55. Here, the phosphorylation during glucose to malate shift increases in occupancy in the Δ*yeaG* strain. In this case, the most logical explanation is that YeaG inactivates another kinase, which is directly responsible for phosphorylating this tyrosine residue. Such regulatory interactions among serine/threonine and tyrosine kinases have been documented in bacteria (Shi et al., 2014) and are most likely at play here. In addition to the proteins with altered SILAC ratios (Table 2), our phosphoproteome study also identified 13 phosphorylation sites detectable in the wild type strain (some of them differentially phosphorylated during the glucose to malate shift) that were no longer phosphorylated at all in the Δ*yeaG* strain (Table 3). Based on the argumentation presented above, these proteins are also very likely to be direct phosphorylation substrates of YeaG.

**Table 2:** List of phosphorylation sites quantified in both strains in both conditions.

Protein name	Phosphorylation site	$\frac{\mathrm{WT}\;\mathrm{malate}}{\mathrm{WT}\;\mathrm{glucose}}$	$\frac{\Delta yeaG\;\mathrm{malate}}{\Delta yeaG\;\mathrm{glucose}}$	$\frac{\left(\frac{\Delta yeaG\;\mathrm{malate}}{\Delta yeaG\;\mathrm{glucose}}\right)}{\left(\frac{\mathrm{WT}\;\mathrm{malate}}{\mathrm{WT}\;\mathrm{glucose}}\right)}$
GlyA	Y55	−3.39	7.58	10.97
AceB	T467	−0.60	5.01	5.61
SerA	S61	−0.42	4.11	4.53
Mdh	T211	−0.34	3.66	4.00
AceA	T5	0.21	3.91	3.70
PykA	S350	−0.55	3.01	3.57
CysK	S133	0.26	3.71	3.45
KdsA	S65	0.10	3.30	3.20
KdsA	S64	0.10	3.10	2.99
TpiA	T179	0.42	3.31	2.89
GpmA	Y92	0.00	2.78	2.78
Pnp	S652	−0.68	2.08	2.76
Pgk	S86	−0.77	1.95	2.72
RpoA	T22	0.54	2.69	2.15
TpiA	S177	0.48	2.53	2.04
FusA	S692	0.50	2.03	1.53
PurM	S191	−0.26	1.26	1.52
FbaA	S268	0.57	1.91	1.34
FolE	S136	−0.41	0.83	1.23
Fbp	S264	1.53	2.69	1.17
InfB	T713	0.13	0.81	0.69
YgaU	S108	1.21	1.88	0.66
OmpF	Y44	0.79	1.44	0.65
CysK	S289	1.55	2.18	0.63
MglA	S406	−2.36	−1.76	0.60
Pgi	T26	−0.66	−0.20	0.46
DnaK	T417	1.58	1.83	0.25
GatY	T233	2.42	2.67	0.24
Pgm	S146	0.11	0.27	0.16
RsxG	T174	−0.12	−0.09	0.03
Hfq	T49	1.45	1.48	0.03
GlnA	Y398	−0.39	−0.65	−0.26
PpsA	T413	1.82	1.13	−0.69
OmpT	S284	0.76	0.02	−0.75
Tsf	S7	1.99	0.85	−1.15
YjhQ	T11	−0.31	−2.14	−1.83

The ratios (log2 transformed) between the level of phosphorylation in different strains and different growth conditions are provided.

**Table 3:** List of proteins identified as phosphorylated in both conditions in the wild type strain but not in the Δ*yeaG* strain.

Protein function	Protein name	Phosphorylation site	$\frac{\mathrm{WT}\;\mathrm{malate}}{\mathrm{WT}\;\mathrm{glucose}}$
Aconitate hydratase 2	AcnB	S244	1.20
Isocitrate lyase	AceA	S398	0.94
DNA protection during starvation protein	Dps	S106	0.71
Superoxide dismutase [Fe]	SodB	T34	0.69
Sensor histidine kinase DpiB	DpiB	S66	0.41
UPF0234 protein YajQ	YajQ	S104	0.29
Isocitrate lyase	AceA	T3	0.22
Sensor histidine kinase DpiB	DpiB	S69	0.22
Sensor histidine kinase DpiB	DpiB	S72	0.22
DNA-directed RNA polymerase subunit alpha	RpoA	S21	0.04
Phosphoenolpyruvate carboxykinase [ATP]	PckA	S250	−0.17
Phosphoribosylformylglycinamidine cyclo-ligase	PurM	S195	−0.42
UPF0339 protein YegP	YegP	T37	−0.53
D-3-phosphoglycerate dehydrogenase	SerA	T63	−1.07
Protein ElaB	ElaB	S60	−1.36

The ratios (log2 transformed) between the level of phosphorylation in different growth conditions are provided.

### YeaG Phosphorylates Isocitrate Lyase AceA

**Figure 3:**
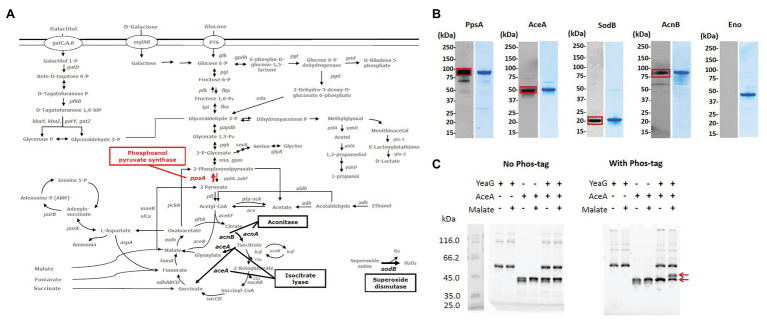
Putative substrates of YeaG that are potentially involved in the Δ*yeaG* phenotyope during glucose to malate shift. **(A)** Proteins that are either less phosphorylated or completely dephosphorylated in Δ*yeaG* strain during glucose to malate shift, mapped on the central carbon metabolism of *E. coli* K-12 MG1655. Red arrows signify phosphorylation events differentially downregulated in Δ*yeaG*, while black arrows indicate phosphorylation events detected only in the wild type strain and undetected in Δ*yeaG*. **(B)** Sodium dodecyl sulphate–polyacrylamide gel electrophoresis (SDS-PAGE) and Western blot images of proteins purified from *E. coli*. Purified N-terminally His-tagged proteins were probed with anti-phospho-serine and anti-phospho-threonine antibodies, according the phosphorylated residue identified in the SILAC experiment. Positive Western signals are highlighted with red squares. Sizes of examined proteins are: PpsA 87.4 kDa, AceA 48.9 kDA, AcnB 93.5 kDa, SodB 21.3 kDa, and Eno (45.7 kDa). AceA, PpsA, SodB, and Eno were probed with the anti-phospho-threonine antibody, while AcnB was probed with the anti-phospho-serine antibody. Eno was used as a negative control. **(C)** AceA is phosphorylated by YeaG *in vitro*. *In vitro* phosphorylation assay was performed with 6 μg of YeaG (74.5 kDa) and 5 μg of AceA (48.9 kDa), in a 20 μl reaction containing 50 mM HEPES pH 7.4, 10 mM MgCl_2_, 10 mM MnCl_2_, 100 mM KCl, 10 mM ATP, 0.1% triton-100, and 10 mM malate, and incubated at 37°C for 2 h. Presence of key components (YeaG, AceA, and malate) in the reactions is indicated with +/− above each lane. After the reaction, proteins were separated by SDS-PAGE, with and without Phos-tag (Fujifilm). Bands corresponding to AceA non-phosphorylated and phosphorylated forms are indicated by red arrows.

In order to assess which potential substrates of YeaG could be involved in the Δ*yeaG* phenotype during glucose to malate shift, in Figure 3A, we mapped them onto the map of *E. coli* central carbon metabolism. Four enzymes feature in this analysis: phosphoenolpyruvate synthase PpsA (phosphorylated at threonine 413), isocitrate lyase AceA (phosphorylated at threonine 3), aconitate hydratase AcnB (phosphorylated at serine 244), and superoxide dismutase SodB (phosphorylated atthreonine 34). To verify the mass spectrometry results that indicated these proteins to be phosphorylated, we first performed a Western blot analysis of the four putative YeaG substrates purified directly from *E. coli*, by using anti-phospho-serine and anti-phospho-threonine antibodies (Figure 3B). All four proteins were confirmed as being phosphorylated to some extent *in vivo*, with somewhat variable intensity of Western signals. Next, we asked whether YeaG is directly responsible for phosphorylating any of these proteins. To verify that, we used Phos-tag, a reagent that can specifically trap phosphorylated proteins, resulting in their lower mobility in SDS-PAGE. In this assay, only AceA tested positive for direct YeaG-dependent phosphorylation (Figure 3C) and all other putative substrates, SodA, PpsA, and AcnB tested negative (Supplementary Figure 5). Interestingly, YeaG phosphorylated AceA only in the presence of malate. This finding suggests that, during the glucose to malate shift, increasing intracellular concentration of malate can lead to activation of the kinase function of YeaG, which in turn phosphorylates AceA. AceA has been shown to have a negative influence on initial metabolic adaptation to glucose starvation and shift to another carbon source (Maharjan et al., 2005). Interestingly, in a long-term adaptation experiment, this influence of AceA was reverted by extragenic changes (mutations in other genes), rather than mutations of *aceA* itself, suggesting regulatory interactions with other proteins. Our data indicate that YeaG has a similarly negative influence on short-term metabolic adaptation to glucose starvation during the shift to malate. Also, our results indicate that YeaG phosphorylates AceA in the presence of malate. Based on this, one could hypothesize that YeaG exerts its negative influence, at least partly, *via* phosphorylation of AceA, which results in prolonging the lag phase during glucose to malate shift. This negative influence can be alleviated either by knocking out the kinase gene itself, as reported in our study, or by inactivating the gene encoding its substrate AceA (Maharjan et al., 2005). During metabolic adaptation to glucose starvation, AceA is known to work in tandem with malate synthase AceB (Maharjan et al., 2005), which is consistent with malate being the intracellular signal for activating the kinase function of YeaG. However, examination of the primary structure of YeaG indicated no known malate binding site. Therefore, the mechanism of this putative interaction of the kinase with malate remains elusive.

### Concluding Remarks and Perspectives

In this study, we have demonstrated that a SILAC-based proteome and phosphoproteome analysis, when implemented under proper physiological conditions, can be a powerful tool in identifying relevant substrates for bacterial protein kinases. The key to success is choosing conditions in which a kinase knockout has a significant phenotype. Herein, we demonstrated that inactivation of the serine/threonine kinase YeaG leads to shortening the lag phase of *E. coli* growth during transition from glucose to malate as the main carbon source. Under those conditions, several proteins were found to be differentially phosphorylated in the Δ*yeaG* strain. By focusing on metabolic enzymes potentially involved in central carbon metabolism, we narrowed down our search for putative YeaG substrates. Then, by using *in vitro* phosphorylation assays, we identified AceA as the substrate of YeaG, which gets phosphorylated specifically in the presence of malate. There is currently not enough evidence to firmly establish the exact mechanism of this newly observed regulatory phenomenon. Further biochemical studies will be needed to clarify the structural and functional aspects of YeaG activation by malate, and the impact of YeaG-dependent phosphorylation on AceA activity. Moreover, physiological studies, e.g., with phospho-mimetic and phospho-ablative mutants of *aceA* will be required to establish a clear link between YeaG-dependent phosphorylation of AceA and the observed lag phase phenotype. Before such studies are carried out, it is hard to make any definite conclusions as to the physiological relevance of YeaG-dependent phosphorylation of AceA.

## Data Availability Statement

The raw data supporting the conclusions of this article will be made available by the authors, without undue reservation.

## Author Contributions

AS performed the strain construction and phenotyping experiments. AS and TG performed and analyzed the proteomics experiments. AS, CJ, MS, JK, and LS performed the protein purification, Western blotting, and phosphorylation experiments. BM assisted in analysis of proteomics experiments. IM supervised the whole study, assisted with data analysis, and wrote the manuscript. All authors contributed to the article and approved the submitted version.

### Conflict of Interest

The authors declare that the research was conducted in the absence of any commercial or financial relationships that could be construed as a potential conflict of interest.
