# Supplementary material for: Phosphoproteome Study of Escherichia coli Devoid of Ser/Thr Kinase YeaG During the Metabolic Shift From Glucose to Malate
Source: Front Microbiol. 2021 Apr 6;12:657562. doi: 10.3389/fmicb.2021.657562 (PMC8055822; doi:10.3389/fmicb.2021.657562)
Supplement: Supplementary file 2 [file Table_2.DOCX]

Supplementary Material

*Escherichia coli* Ser/Thr kinase YeaG regulates the metabolic shift from glucose to malate by phosphorylating isocitrate lyase AceA

Abida Sultan^1^, Carsten Jers^1^, Lei Shi^2^, Meriem Senissar^1^, Tariq A. Ganief^3^, Boris Macek^3^, Ivan Mijakovic^1,2,#^

^1^ Novo Nordisk Foundation Center for Biosustainability, Technical University of Denmark, Kemitorvet 220, DK-2800 Kgs. Lyngby, Denmark

^2^ Systems and Synthetic Biology Division, Department of Biology and Biological Engineering, Chalmers University of Technology, Kemivägen 10, SE-412 96 Gothenburg, Sweden

^3^ Quantitative Proteomics and Proteome Center Tuebingen, Interfaculty Institute for Cell Biology, University of Tuebingen, Auf der Morgenstelle 15, 72076 Tuebingen, Germany

# Correspondence:

Prof. Ivan Mijakovic

ivan.mijakovic@chalmers.se

# Supplementary Tables

## Supplementary Table 1. Total list of proteins and phospho-sites detected by MS proteomics. MS data sheets for proteomic and phosphoproteomic experiments, a list of differentially regulated proteins and phospho-proteins as well as GO term analysis data are provided. (Available as a separate Excel sheet.)

## Supplementary Figures


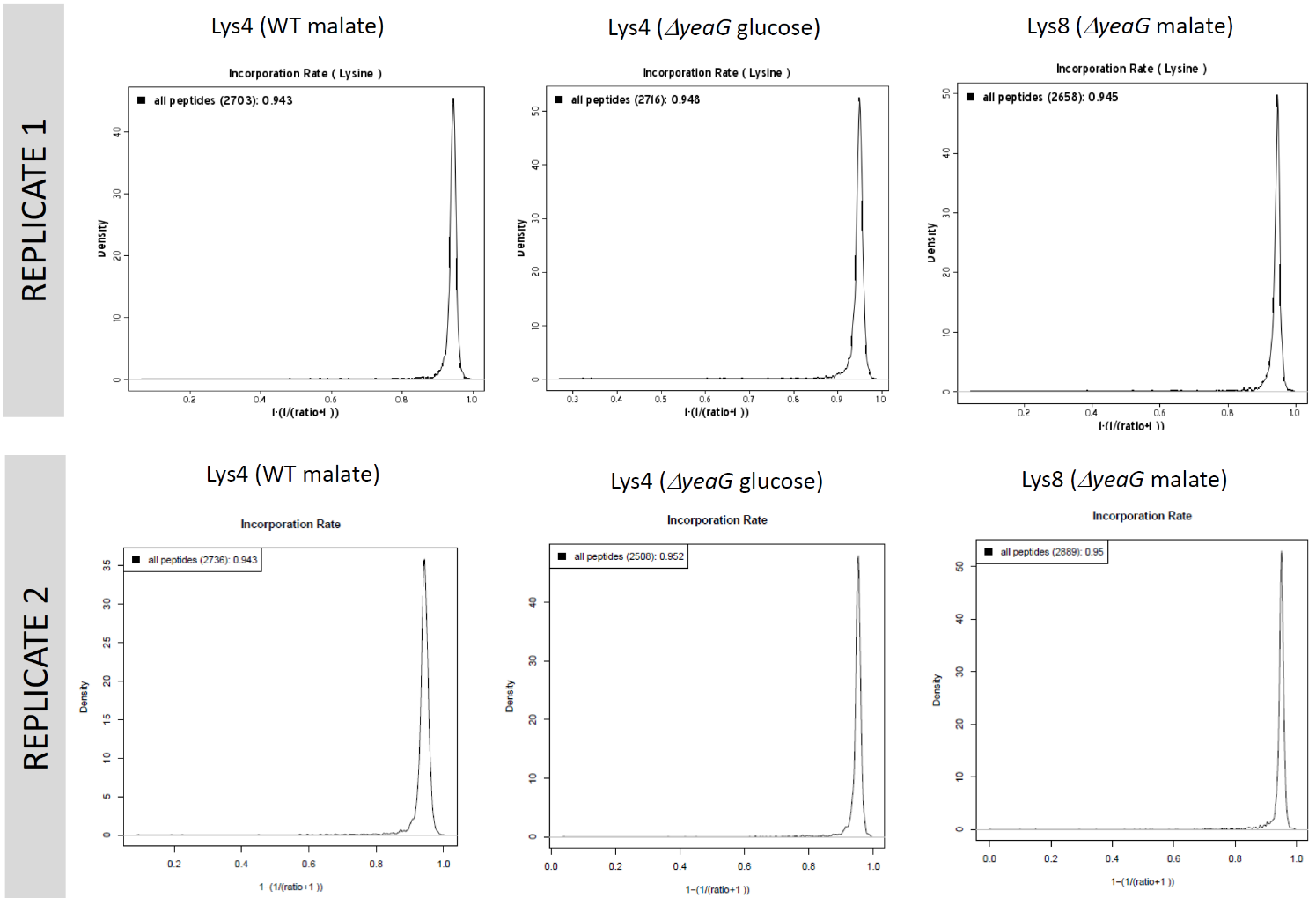


**Supplementary Figure 1.** SILAC amino acid (Lys4 and Lys8) incorporation levels in both biological replicates. In all samples, heavy lysine incorporation level was > 94%.


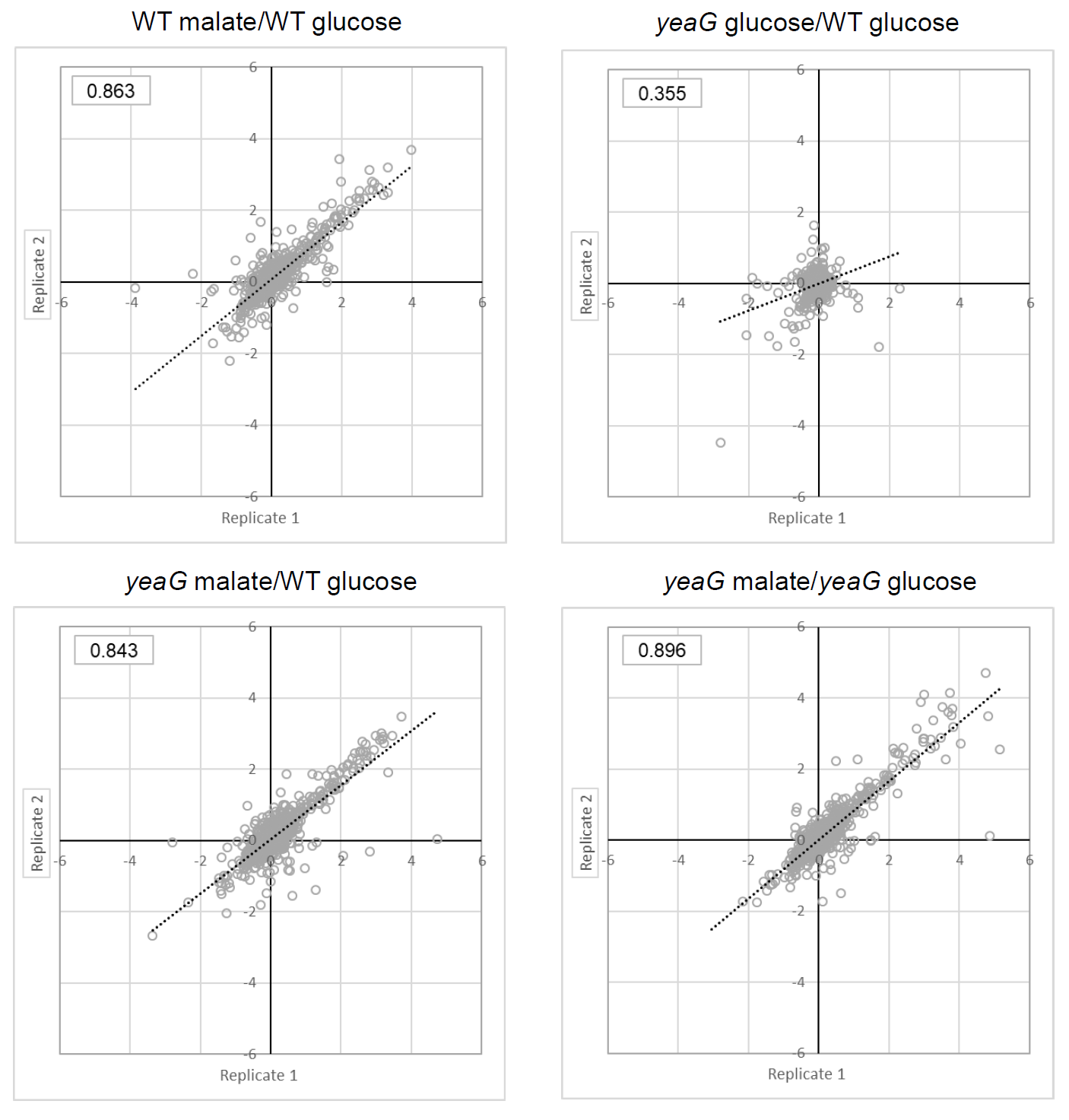


**Supplementary Figure 2.** Reproducibility of SILAC quantification. Correlation of protein groups between the two biological replicates. Calculated Pearson correlation coefficient is indicated on each plot.


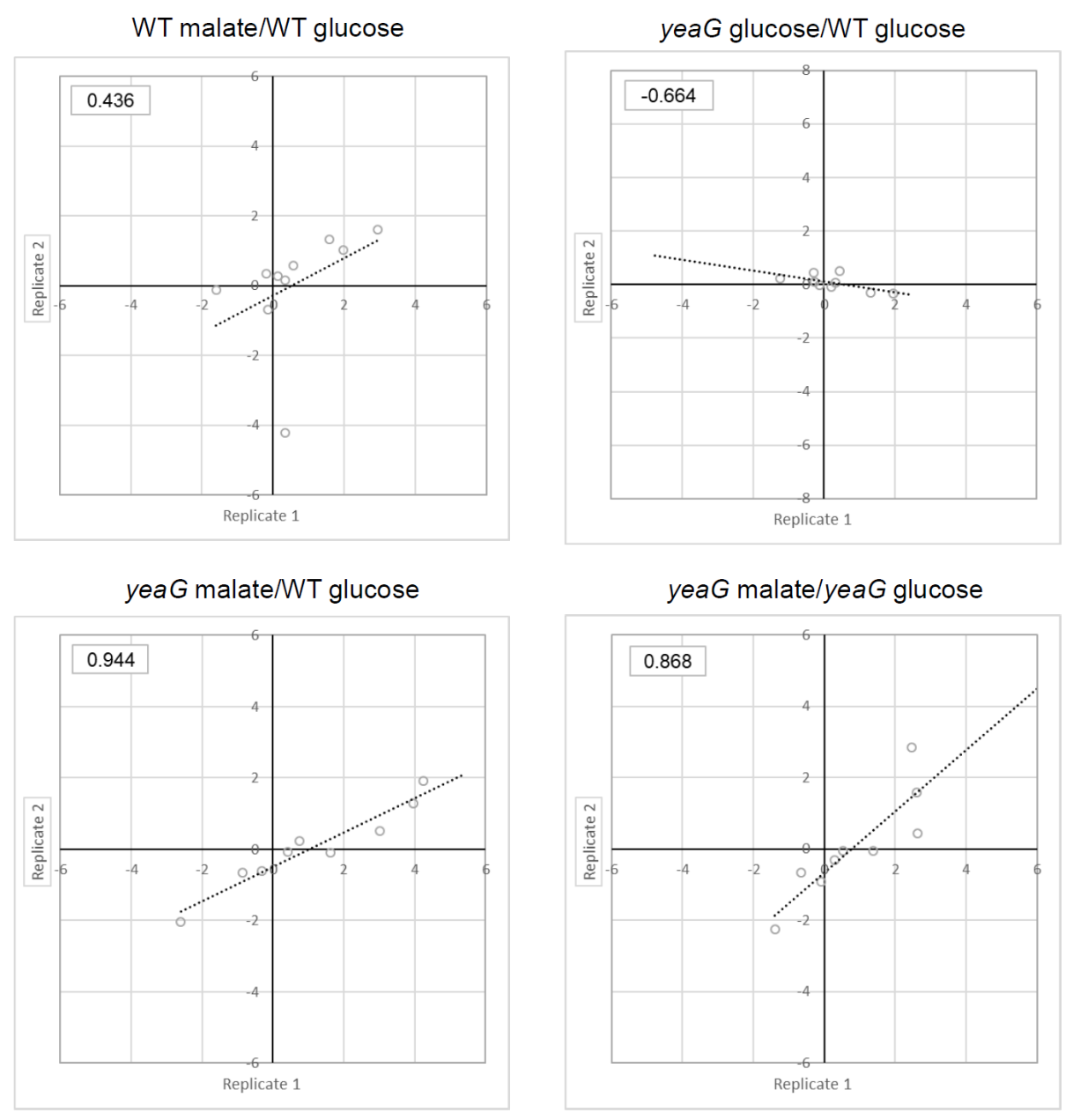


**Supplementary Figure 3.** Reproducibility of SILAC quantification. Correlation of phosphorylation sites between the two biological replicates. Calculated Pearson correlation coefficient is indicated on each plot.

|  |
| --- |
|  |
|  |

**Supplementary Figure 4.** Bar charts depicting functional annotation analysis of differentially expressed proteins. Enriched GO terms for biological process (GOBP), molecular function (GOMF) and cellular component (GOCC) that were significant (*p*-value < 0.05 after correcting for multiple testing by Bonferroni false discovery rate) are shown for each strain: wild type is shown in black and Δ*yeaG* in grey. Up to 40 most significant terms are depicted, for a full list of GO terms see supplementary Table S1.

**Supplementary Figure 5.** *In vitro* phosphorylation assays were performed with 6 µg of YeaG (74.5 kDa) and 5 µg of each of its putative substrate proteins (protein names indicated above each gel), in a 20 µl reaction containing 50 mM HEPES pH 7.4, 10 mM MgCl2, 10 mM MnCl2, 100 mM KCl, 10 mM ATP, 0.1% triton-100 and 10 mM malate, and incubated at 37 °C for 2 h. Presence of key components (YeaG, protein substrates and malate) in the reactions is indicated with +/- above each lane. After the reaction, proteins were separated by SDS-PAGE, with and without Phos-tag (Fujifilm). A) gels not treated with Phos-tag. B) Gels treated with Phos-tag.
